# Contribution of the Twin-Arginine Translocation System to the Intracellular Survival of *Salmonella* Typhimurium in *Dictyostelium discoideum*

**DOI:** 10.3389/fmicb.2018.03001

**Published:** 2018-12-06

**Authors:** Ítalo M. Urrutia, Andrea Sabag, Camila Valenzuela, Bayron Labra, Sergio A. Álvarez, Carlos A. Santiviago

**Affiliations:** Laboratorio de Microbiología, Departamento de Bioquímica y Biología Molecular, Facultad de Ciencias Químicas y Farmacéuticas, Universidad de Chile, Santiago, Chile

**Keywords:** *Salmonella*, *Dictyostelium*, Tat system, infection, intracellular survival, social development, virulence

## Abstract

The twin-arginine translocation (Tat) system is a specialized secretion pathway required for bacteria to export fully folded proteins through the cytoplasmic membrane. This system is crucial during *Salmonella* infection of animal hosts. In this study, we show that *Salmonella enterica* serovar Typhimurium (*S*. Typhimurium) requires the Tat system to survive and proliferate intracellularly in the social amoeba *Dictyostelium discoideum*. To achieve this, we developed a new infection assay to assess intracellular bacterial loads in amoeba by direct enumeration of colony forming units (CFU) at different times of infection. Using this assay we observed that a Δ*tatABC* mutant was internalized in higher numbers than the wild type, and was defective for intracellular survival in the amoeba at all times post infection evaluated. In addition, we assessed the effect of the Δ*tatABC* mutant in the social development of *D*. *discoideum*. In contrast to the wild-type strain, we observed that the mutant was unable to delay the social development of the amoeba at 2 days of co-incubation. This phenotype correlated with defects in intracellular proliferation presented by the Δ*tatABC* mutant in *D*. *discoideum* after 24 h of infection. All phenotypes described for the mutant were reverted by the presence of a plasmid carrying *tatABC* genes, indicating that abrogation of Tat system attenuates *S*. Typhimurium in this model organism. Overall, our results indicate that the Tat system is crucial for *S*. Typhimurium to survive and proliferate intracellularly in *D. discoideum* and for virulence in this host. To the best of our knowledge, this is the first report on the relevance of the Tat system in the interaction of any bacterial pathogen with the social amoeba *D. discoideum*.

## Introduction

The genus *Salmonella* includes species *S. enterica* and *S. bongori*, which can be differentiated into more than 2,500 serovars according to variations of surface antigens. Some serovars within *S. enterica*, such as *Salmonella enterica* serovar Typhimurium (*S*. Typhimurium), are named generalist as they can infect a wide range of hosts, causing illnesses ranging from gastroenteritis to severe systemic disease (Tsolis et al., 1999; Zhang et al., 2003). These pathogens are highly versatile and can adapt to a variety of conditions both in the natural environment and within a host.

Internalization and intracellular survival in host cells are essential processes for *Salmonella* virulence that depend on the translocation of bacterial effector proteins to eukaryotic cells through the type 3 secretion systems (T3SS) encoded in SPI-1 and SPI-2 (T3SS_SPI-1_ and T3SS_SPI-2_, respectively) (Haraga et al., 2008). In addition, it has been reported that other secretion systems, such as the twin-arginine translocation (Tat) system, are required for *Salmonella* infection (Mickael et al., 2010; Reynolds et al., 2011; Craig et al., 2013).

The Tat system is found in most bacteria and has the unusual property of transporting fully folded proteins from the cytoplasm to the periplasmic space (Palmer and Berks, 2012). In *Escherichia coli* and *Salmonella*, the main components of this system are proteins TatA, TatB, and TatC. TatB and TatC form an integral membrane complex that recognizes substrates containing an amino-terminal signal peptide that includes the twin-arginine consensus motif S/T-R-R-x-F-L-K. Once the substrate has been recognized, the TatBC complex induces the polymerization of TatA to form the pore that allows the passage of the fully folded protein through the cytoplasmic membrane (Palmer and Berks, 2012).

It has been described that inactivation of genes encoding Tat components in several bacteria including *E. coli*, *Legionella pneumophila*, *Yersinia pseudotuberculosis*, *Mycobacterium smegmatis*, *Vibrio cholerae*, *Pseudomonas aeruginosa* and *S.* Typhimurium results in defects in several bacterial properties and processes, including cell morphology, growth and biofilm formation, cell wall integrity, transport of virulence factors, cell division, motility and chemotaxis (Bogsch et al., 1998; Santini et al., 1998; Voulhoux et al., 2001; Ochsner et al., 2002; Ding and Christie, 2003; Pradel et al., 2003; Harrison et al., 2005; McDonough et al., 2005; Rossier and Cianciotto, 2005; Lavander et al., 2006; Posey et al., 2006; Zhang et al., 2009; Reynolds et al., 2011). Regarding the role of the Tat system in *Salmonella* virulence, it has been reported that a Δ*tatC* mutant of *S.* Typhimurium presents colonization defects in mice (Santiviago et al., 2009; Reynolds et al., 2011; Craig et al., 2013) and reduced intracellular replication in J774-A1 murine macrophages (Reynolds et al., 2011). In addition, inactivation of genes *tatB* and *tatC* in *Salmonella* Enteritidis results in impaired Caco-2 cell invasion and reduced colonization in mice and chickens (Mickael et al., 2010; Silva et al., 2012).

During its life cycle outside animal hosts, *Salmonella* interacts with a wide variety of predatory eukaryotic organisms in the environment, including amoeba. Amoeba are organisms that feed on fungi and bacteria by phagocytosis (Salah et al., 2009; Denoncourt et al., 2014; Hoffmann et al., 2014). Several bacterial pathogens have been described to survive predation by these organisms, including *L. pneumophila*, *Mycobacterium marinum*, *Bordetella bronchiseptica*, *P. aeruginosa* and *V. cholerae*, among others (Abd et al., 2005, 2007; Cardenal-Muñoz et al., 2017; Strassmann and Shu, 2017; Taylor-Mulneix et al., 2017; Swart et al., 2018). Recently, we and other researchers have reported that *S*. Typhimurium can survive within the social amoeba *Dictyostelium discoideum* (Sillo et al., 2011; Riquelme et al., 2016; Varas et al., 2018) and requires T3SS_SPI-1_ and T3SS_SPI-2_ for this process, among other virulence factors (Riquelme et al., 2016).

In this work, we assessed the contribution of the Tat system to the interaction of *S.* Typhimurium with *D. discoideum*. To this end, we developed a CFU-based infection assay using axenic *D*. *discoideum* to evaluate the intracellular survival of wild-type and Δ*tatABC* strains in the amoeba. Our results showed that the Δ*tatABC* mutant presents intracellular survival and proliferation defects during infection in this host. In addition, we observed that the Δ*tatABC* mutant was unable to delay the social development of the amoeba caused by the wild-type strain. Altogether, our results indicate that the Tat system plays a major role during the interaction of *S*. Typhimurium with *D*. *discoideum*.

## Materials and Methods

### Bacterial Strains, Media and Culture Conditions

Bacterial strains used in this study are described in Table 1. All *S*. Typhimurium strains are derivatives of the wild-type, virulent strain 14028s (Fields et al., 1986; Jarvik et al., 2010). Bacteria were routinely grown statically in Luria-Bertani (LB) medium (10 g/L tryptone, 5 g/L NaCl and 5 g/L yeast extract) at 37°C. When required, the medium was supplemented with ampicillin (100 mg/L), chloramphenicol (20 mg/L) or kanamycin (75 mg/L). Media were solidified by the addition of agar (15 g/L). All procedures involving the use of pathogenic organisms were conducted following the guidelines in the Biosafety Manual (2018 version) of the National Commission of Scientific and Technological Research (CONICYT, Chile), and were approved by the Biosafety Committee of Universidad de Chile, Campus Norte.

**Table 1:** Bacteria and *Dictyostelium* strains used in this study.

Strain	Characteristic or genotype	Source or reference
***Salmonella* Typhimurium**		
WT	Wild-type, virulent strain 14028s	Laboratory collection (Fields et al., 1986; Jarvik et al., 2010)
Δ*tatABC*	Δ*tatABC*::Kan	This study
Δ*tatABC*/pTAT	Δ*tatABC*::Kan transformed with plasmid pBAD-TOPO::*tatABC*	This study
Δ*tatABC*/pBAD	Δ*tatABC*::Kan transformed with plasmid pBAD-TOPO	This study
Δ*aroA*	Δ*aroA*::Kan	Laboratory collection (Varas et al., 2017)
Δ*phoN*	Δ*phoN*::Cam	Laboratory collection (Riquelme et al., 2016)
***Escherichia coli***		
B/r (DBS0348878)	Wild-type strain	Dicty Stock Center (dictyBase)
***Klebsiella aerogenes***		
DBS0305928	Wild-type strain	Dicty Stock Center (dictyBase)
***Dictyostelium discoideum***		
AX4 (DBS0302402)	*axeA1* *axeB1* *axeC1*	Dicty Stock Center (dictyBase)


### *Dictyostelium* Strains, Media and Culture Conditions

*D. discoideum* strain AX4 (DBS0302402) (Table 1) was obtained from Dicty Stock Center (Basu et al., 2013; Fey et al., 2013), and cultured according to standard protocols (Fey et al., 2007). Briefly, amoeba were maintained at 22°C in SM agar (10 g/L glucose, 10 g/L peptone, 1 g/L yeast extract, 1 g/L MgSO_4_ × 7H_2_O, 1.9 g/L KH_2_PO_4_, 0.6 g/L K_2_HPO_4_, 20 g/L agar), growing on a confluent lawn of *Klebsiella aerogenes* (DBS0305928). For infection assays, amoeba were grown axenically at 22°C in HL5 medium (14 g/L tryptone, 7 g/L yeast extract, 0.35 g/L Na_2_HPO_4_, 1.2 g/L KH_2_PO_4_, 14 g/L glucose, pH 6.3) with agitation at 180 rpm. When required, HL5 medium was supplemented with a mix of streptomycin (300 mg/L) and ampicillin (100 mg/L). Prior to infection, amoeba were harvested at the early exponential phase (1–2 × 10^6^ cells/mL) and centrifuged at 300 ×*g* for 5 min at 4°C. The supernatant was discarded and the pellet was washed three times using Soerensen buffer (2 g/L KH_2_PO_4_, 0.36 g/L Na_2_HPO_4_ × 2H_2_O, pH 6.0). Trypan blue exclusion and counting in a Neubauer chamber was used to determine the population of viable cells.

### Construction of Mutant Strains

A *S*. Typhimurium Δ*tatABC* mutant strain was constructed using the Lambda Red recombination method (Datsenko and Wanner, 2000) with modifications (Santiviago et al., 2009), using primers tatABC_(H1+P1) and tatABC_(H2+P2), and plasmid pCLF4 (Kan^R^, GenBank accession number EU629214) as template for PCR amplification. The correct allelic replacement in this mutant was confirmed by PCR amplification using primers tatABC_Out5 and tatABC_Out3, flanking the substitution site. All primers for PCR amplifications are listed in Table 2.

**Table 2:** Primers used in this study.

Primer name	Sequence
tatABC_(H1+P1)	AGGAACATGTATGGGTGGTATCAGTATTTGGCAGTTGTTGGTGCAGGCTGGAGCTGCTTC
tatABC_(H2+P2)	GCGGTTGTGTTTAGTCTTCAGTGTGCTCGGCCTTTTCGGTCATATGAATATCCTCCTTAG
tatABC_Out5	GAGCGGGTCATTCTTACTCG
tatABC_Out3	TTCGTTCCGGTCAGTAGCAT
pBAD_Forward	ATGCCATAGCATTTTTATCC
pBAD_Reverse	GATTTAATCTGTATCAGG

*Underlined sequences anneal to the 5′ or 3′ end of the antibiotic-resistance cassette in template vector pCLF4 (Kan^R^, GenBank accession number EU629214).*

### Construction of Complementing Plasmid pTAT

A DNA fragment containing genes *tatABC* (including the promoter region) was amplified from the genome of *S*. Typhimurium strain 14028s using *Taq* DNA polymerase (Invitrogen) and primers tatABC_Out5 and tatABC_Out3 (Table 2). The PCR product was purified from 1% agarose gels using the “QIAquick Gel Extraction Kit” (QIAGEN) and cloned into pBAD-TOPO using the “pBAD-TOPO TA Expression Kit” (Invitrogen). The presence and orientation of the insert in the recombinant plasmid generated (pTAT) was confirmed by PCR amplification using combinations of primers tatABC_Out5, tatABC_Out3, pBAD_Forward and pBAD_Reverse (Table 2). The *S*. Typhimurium Δ*tatABC* mutant was transformed by electroporation with plasmids pTAT or pBAD-TOPO for complementation assays.

### Individual Infection Assay

An infection assay was developed (Supplementary Figure S1) based on a method previously described by our group (Riquelme et al., 2016). Each bacterial strain to be evaluated was grown overnight, suspended in LB medium adjusting the OD_600nm_ to 0.2, harvested and washed two times with Soerensen buffer. Next, 150 μL of each bacterial suspension was further diluted by adding 850 μL of Soerensen buffer. In parallel, *D. discoideum* AX4 cells from axenic cultures grown to exponential phase were washed two times with Soerensen buffer, and titrated by Trypan blue exclusion and counting on a Neubauer chamber. Next, a suspension containing ∼1 × 10^6^ amoeba/mL was prepared using Soerensen buffer. Aliquots of 100 μL from bacteria and amoeba suspensions were mixed in an Eppendorf tube in order to obtain a multiplicity of infection (MOI) of ∼100 bacteria/amoeba. The mixture was centrifuged at 9,200 ×*g* for 20 s (to promote the interaction of bacteria with amoeba), and incubated at 22°C for 1 h. After the incubation, all mixes were centrifuged at 300 ×*g* for 5 min, washed once with Soerensen buffer supplemented with gentamicin (10 mg/L) to kill extracellular bacteria, then centrifuged and washed twice with Soerensen buffer to remove the antibiotic, and finally centrifuged and suspended in Soerensen buffer (final volume: 100 μL). The content of two tubes carrying a suspension of infected amoeba after 0, 1, 3, 5, 10 or 24 h post infection was analyzed in parallel to determine viable amoeba and intracellular bacteria. Viable amoeba in the first tube were determined at each time point by Trypan blue exclusion and counting on a Neubauer chamber. In addition, infected amoeba recovered from the second tube were washed once with Soerensen buffer and finally lysed with 0.2% Triton X-100. Titers of intracellular bacteria were determined by serial dilutions and plating on LB agar.

### Bacterial Adherence Assay

A procedure similar to our infection assay was implemented to evaluate adherence of different bacterial strains to *D. discoideum* AX4 cells. Briefly, suspensions of bacteria and amoeba were prepared as indicated above and mixed at a MOI of ∼100 bacteria/amoeba in an Eppendorf tube. Next, each mixture was centrifuged (9,200 ×*g* for 20 s) to promote the interaction of bacteria with amoeba, and further incubated at 4°C for 30 min. After the incubation, all mixtures were centrifuged (300 ×*g* for 5 min) at 4°C and the infected amoeba were washed 3 sequential times with Soerensen buffer at 4°C to remove non-adhered bacteria. Finally, amoeba were lysed with 0.2% Triton X-100 and titers of adhered bacteria were determined by serial dilutions and plating on LB agar.

### Bacterial Toxicity Assay

A modified version of the virulence assay reported by Champion and colleagues (Champion et al., 2016) was implemented to indirectly evaluate bacterial cytotoxicity toward *D. discoideum* evidenced by changes in cell morphology of amoeba co-incubated with different *S*. Typhimurium strains and *E. coli* B/r. Briefly, bacteria and amoeba were cultured as indicated in the infection assay procedure. Aliquots (100 μL) of a suspension containing ∼1 × 10^6^ amoeba/mL prepared in HL5 medium were added to the wells of a 96-well plate and incubated overnight at 22°C to allow adhesion of amoeba to the bottom of the wells. The next day, the HL5 medium in wells containing adhered amoeba was replaced with aliquots (100 μL) of bacterial suspensions prepared in Soerensen buffer in order to obtain MOIs of 100 or 1,000 bacteria/cell. The plates were incubated at 22°C and the cell shape of infected amoeba in each well was monitored at different co-incubation times (0, 30, 60, 90, 120, 150, and 180 min) using a Motic AE 2000 inverted microscope equipped with a Plan Achromatic Phase LWD PL Ph2 40× objective. Representative images were acquired using a Moticam 580 (5.0 MP) digital camera attached to the trinocular port of the microscope using a C-mount adapter (0.5×).

### Social Development Assay

Individual wells of a 24-well plate containing N agar (Soerensen buffer supplemented with 1 g/L peptone, 1 g/L glucose and 20 g/L agar) were inoculated with 30 μL of an overnight culture from each bacterial strain to be evaluated. The plate was incubated overnight at 22°C to obtain bacterial lawns. The next day, 10 μL of a suspension containing ∼1 × 10^4^ axenic *D. discoideum* AX4 cells in HL5 was spotted in the center of each well on top of the corresponding bacterial lawn and the plate was further incubated at 22°C for 2 days. Representative images of *D. discoideum* development were obtained at days 1 and 2 using a Motic SMZ-171 stereomicroscope equipped with a Moticam 580 (5.0 MP) digital camera attached to the trinocular port of the stereomicroscope using a C-mount adapter (0.5×).

## Results and Discussion

### New Infection Assay to Evaluate Internalization and Intracellular Survival in *D. discoideum*

**FIGURE 1:**
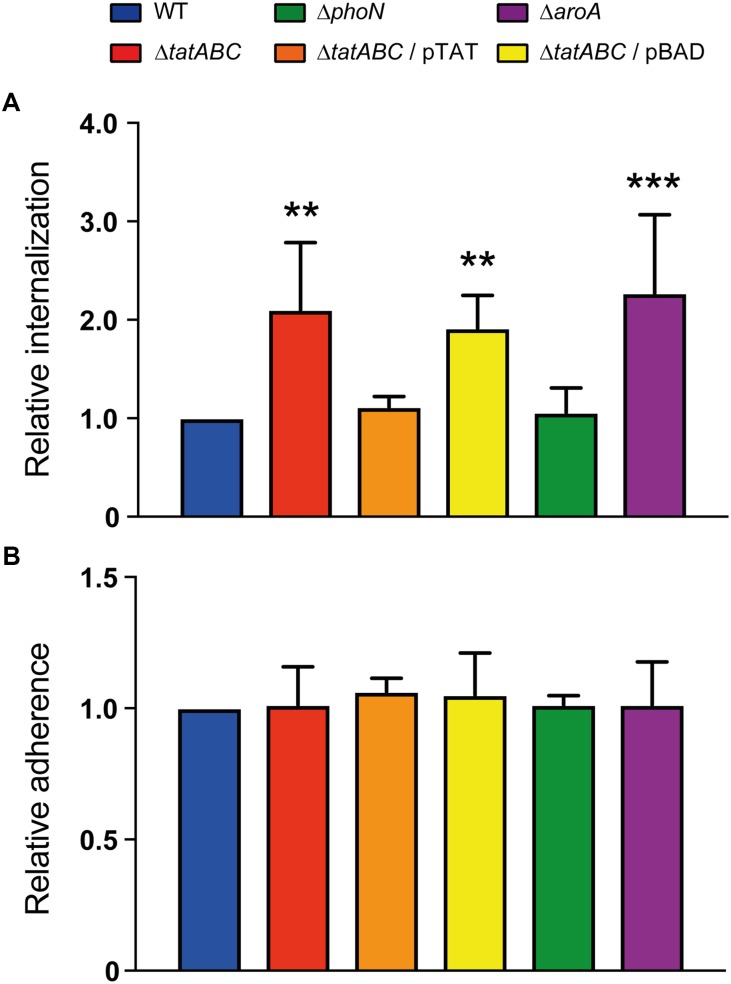
Adherence and internalization of *S*. Typhimurium strains in *D. discoideum*. Individual infection assays were conducted to evaluate the adherence and internalization of different *S*. Typhimurium strains in *D*. *discoideum* AX4. **(A)** Relative internalization after 1 h of infection at 22°C. **(B)** Relative adherence after 30 min of co-incubation at 4°C. All values (internalization and adherence) were calculated as CFU*_t_*_=0_/CFU_inoculum_ and further normalized to the value of the wild-type strain. Graphs show mean values ± SD from at least 7 independent assays. Statistical significance of differences in internalization or adherence between each strain and the wild type was determined using a one-way ANOVA with Dunnett’s test (^∗∗^*P* < 0.01; ^∗∗∗^*P* < 0.001).

To evaluate the role played by the Tat system in the survival of *S*. Typhimurium in *D. discoideum*, we developed a new CFU-based infection assay where amoeba were infected with the wild type and mutant strains, and intracellular bacteria were recovered from infected amoeba and titrated at different times post infection to evaluate internalization and intracellular survival (Supplementary Figure S1). The main advantage of this method is that it uses smaller volumes, and therefore fewer amoeba cells, than assays previously reported by our group (Riquelme et al., 2016; Varas et al., 2018). This simplifies the handling of samples, allowing the analysis of more strains per experiment than assays requiring larger volumes. Although this method is based on a described assay (Riquelme et al., 2016), we used our new infection assay to analyze the wild-type strain and included Δ*aroA* and Δ*phoN* mutants as controls in order to validate our observations. The internalization of each strain was evaluated after 1 h of infection. We observed that the Δ*aroA* mutant was internalized in higher numbers than the wild type, and the Δ*phoN* mutant was internalized at wild-type levels (Figure 1A). In addition, the Δ*aroA* mutant presented defects in intracellular survival in the amoeba while the Δ*phoN* mutant presented wild-type levels of intracellular survival (Figure 3A). These observations are consistent with results from different infection assays showing that Δ*aroA* and Δ*phoN* mutants are attenuated and not attenuated in this model organism, respectively (Riquelme et al., 2016; Varas et al., 2018).

### A *S.* Typhimurium Δ*tatABC* Mutant Is Defective for Intracellular Survival in *D. discoideum*

Once validated, we used our infection assay to evaluate the internalization and intracellular survival of a Δ*tatABC* mutant in *D. discoideum*. We observed that this mutant was internalized at higher levels than the wild-type strain, as in the case of the Δ*aroA* mutant (Figure 1A). To gain further insight into the causes of the observed phenotype, we conducted an assay to evaluate the adherence of our strains to *D. discoideum* cells. We observed that all strains presented identical adherence levels (Figure 1B), indicating that the elevated internalization levels presented by Δ*tatABC* (and other mutants under study) are not attributable to increased adherence to amoeba cells resulting in higher uptake.

**FIGURE 2:**
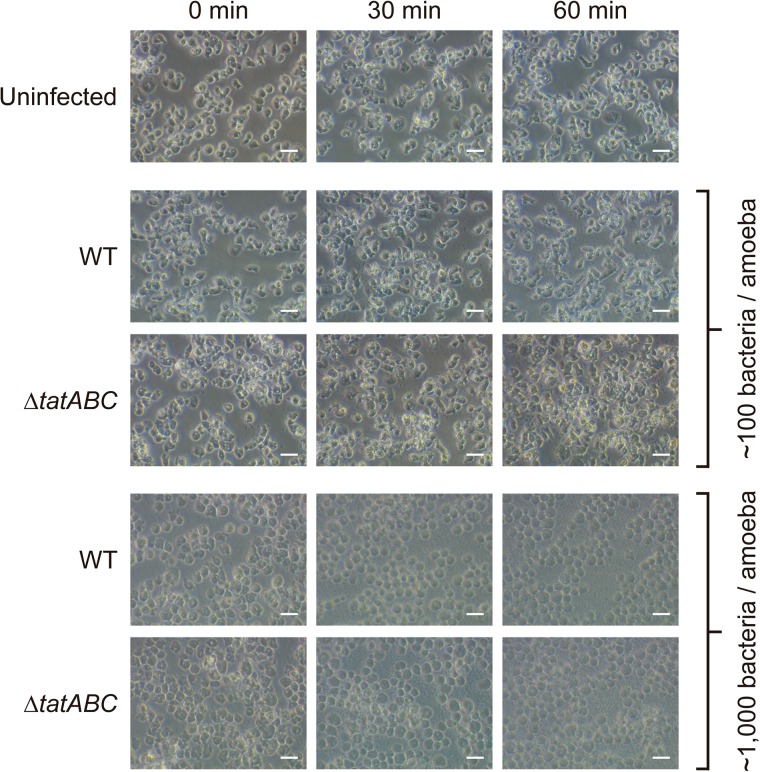
Qualitative evaluation of cytotoxicity caused by *S. Typhimurium* strains on *D. discoideum*. The shape of *D*. *discoideum* AX4 cells infected with different strains of *S*. Typhimurium was monitored at 0, 30, and 60 min of co-incubation at 22°C in Soerensen buffer. Representative images from 3 independent assays carried out at MOIs of ∼100 or ∼1,000 bacteria/amoeba are shown. Scale bar, 20 μm.

Furthermore, we conducted a modified version of an assay originally developed to evaluate virulence of *Pseudomonas aeruginosa* strains during co-incubation with *D. discoideum* in buffer (Champion et al., 2016). The authors of the method reported that virulent strains of *P. aeruginosa* caused cell rounding and cytoplasmic shrinkage of amoeba, that were interpreted as a cytotoxic effect exerted by these strains (Champion et al., 2016). In our case, co-incubations of amoeba with each bacterial strain at a MOI of ∼100 bacteria/amoeba for up to 180 min produced no changes in *D. discoideum* cell shape (Supplementary Figure S2). Figure 2 shows representative results of amoeba co-incubated for up to 60 min with the wild-type strain or the Δ*tatABC* mutant. These results suggest that all bacterial strains evaluated produced no cytotoxic effects to amoeba cells under the experimental conditions evaluated. In contrast, during co-incubations conducted at a MOI of ∼1,000 bacteria/amoeba all strains caused detachment of amoeba from the wells and a rounded cell morphology at all times evaluated (Figure 2 and Supplementary Figure S2), suggesting the existence of a cytotoxic effect toward *D. discoideum* under these experimental conditions. Therefore, most probably the differences in internalization levels presented by each strain under study are not caused by differential cytotoxic effects exerted by these bacteria on amoeba cells under the conditions routinely used in our infection assay (MOI of ∼100 bacteria/amoeba).

**FIGURE 3:**
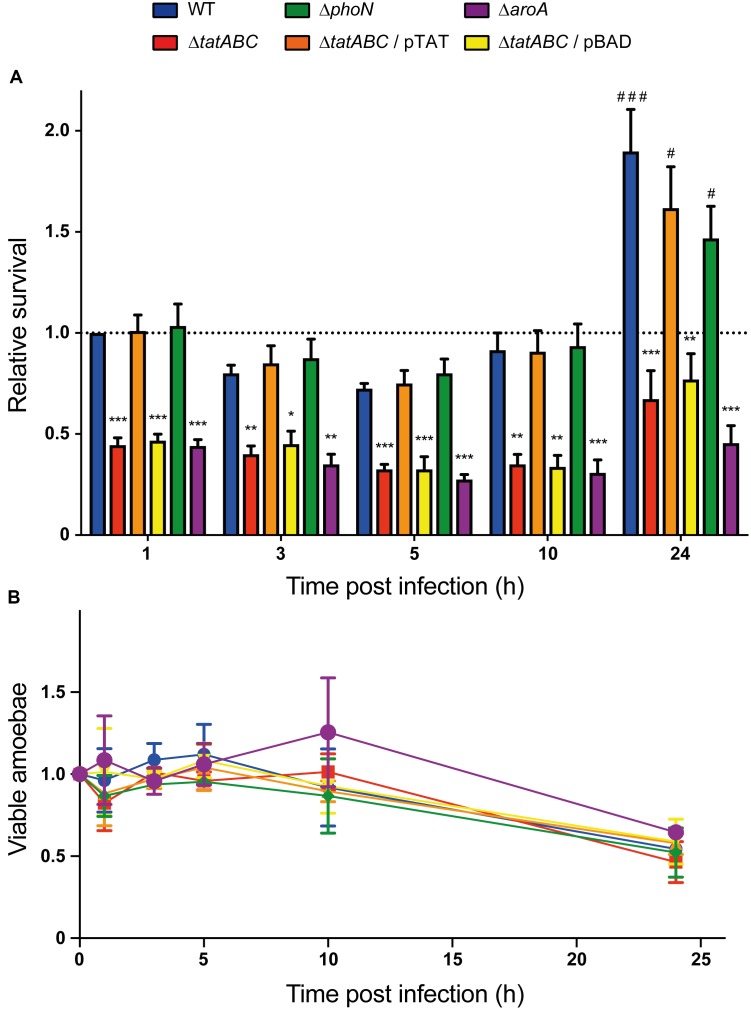
Intracellular survival of *S*. Typhimurium strains in *D. discoideum*. Individual infection assays were conducted to evaluate the intracellular survival of different *S*. Typhimurium strains in *D*. *discoideum* AX4. **(A)** Relative intracellular survival at different times post infection. All values were calculated as CFU*_t_*_=x_/CFU*_t_*_=0_ and further normalized to the value of the wild-type strain at *t* = 1. **(B)** Variation in the population of viable amoeba during the infection assay. All values were calculated as cells/mL and further normalized to the value of amoeba infected with the wild-type strain at *t* = 0. Graphs show mean values ± SD from at least 7 independent assays. Statistical significance of differences in intracellular survival between each strain and the wild type at a given time was determined using a one-way ANOVA with Dunnett’s test (^∗^*P* < 0.05; ^∗∗^*P* < 0.01; ^∗∗∗^*P* < 0.001). Statistical significance of differences in intracellular survival of a given strain at *t* = 1 versus *t* = 24 was determined using a one-way ANOVA with Dunnett’s test (^#^*P* < 0.05; ^###^*P* < 0.001).

In addition to the internalization phenotype revealed by our infection assay, we observed that the Δ*tatABC* mutant was impaired for intracellular survival in *D. discoideum* at all times evaluated, as in the case of the Δ*aroA* mutant (Figure 3A). No effect in amoeba viability was observed during the course of the infections (Figure 3B), indicating that the phenotypes shown by the different strains are not attributable to changes in the number of viable amoeba all through the assay. Of note, all phenotypes presented by the Δ*tatABC* mutant were reverted by the presence of a plasmid carrying genes *tatABC* (pTAT), and the presence of the empty vector (pBAD) did not affect the phenotypes shown by this mutant (Figures 1, 3). During the course of the infection assay we noticed that all strains slightly decreased their intracellular titers during the first 5 h post infection. We also observed intracellular proliferation in the case of the wild-type strain at 24 h post infection. The proliferation level shown by the wild-type strain at this time point was comparable to that shown by strains Δ*phoN* and Δ*tatABC* harboring plasmid pTAT, roughly reaching twice the amount of intracellular bacteria present at 1 h post infection. In contrast, in the case of strains Δ*tatABC*, Δ*tatABC* harboring plasmid pBAD and Δ*aroA* the amount of intracellular bacteria at 24 h post infection was comparable to the levels shown by each strain at 1 h post infection (Figure 3A).

Taken together, our results indicate that *S*. Typhimurium requires the Tat system to survive and proliferate in *D. discoideum*. This is the first report on the relevance of the Tat system in *Salmonella* survival within this amoeba. These observations are consistent with the role reported for Tat in the intracellular survival of *S.* Typhimurium in J774-A1 murine macrophages (Reynolds et al., 2011). Regarding the role played by Tat in the survival of other bacterial pathogens in protozoa, it has been reported that inactivation of genes encoding components of this system in *L. pneumophila* results in intracellular survival defects in *Acanthamoeba castellanii* and *Hartmannella vermiformis* (De Buck et al., 2005; Rossier and Cianciotto, 2005). These studies also revealed that *L. pneumophila* requires Tat for intracellular survival in U937 human monocytes differentiated into macrophage-like cells (De Buck et al., 2005; Rossier and Cianciotto, 2005).

### A *S.* Typhimurium Δ*tatABC* Mutant Is Unable to Delay the Social Development of *D. discoideum*

**FIGURE 4:**
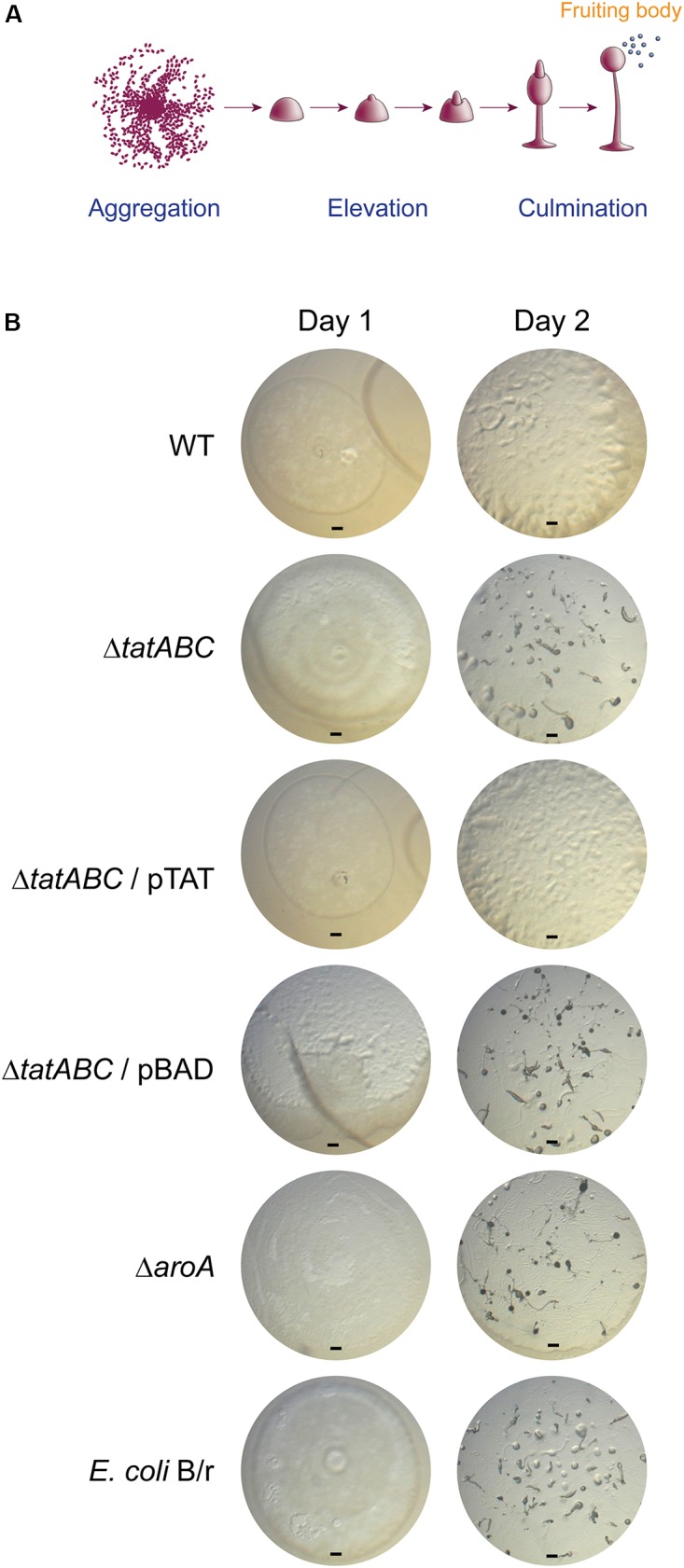
Social development of *D*. *discoideum* co-incubated with *S*. Typhimurium 14028s derivatives and *E. coli* B/r. **(A)** Main phases of the *D*. *discoideum* development cycle, including aggregation, elevation and culmination to generate fruiting bodies (scheme adapted from Fey et al., 2007). **(B)** *D*. *discoideum* development after 1 and 2 days of co-incubation with *S*. Typhimurium strains or *E*. *coli* B/r. Representative images from 3 independent assays are shown. Scale bar, 100 μm.

It has been reported that virulent pathogenic bacteria delay the social development of *D. discoideum*, while attenuated or non-pathogenic bacteria allow its rapid progression (Bravo-Toncio et al., 2016; Ouertatani-Sakouhi et al., 2017; Marcoleta et al., 2018). In fact, wild-type *S*. Typhimurium causes a significant delay in the development of this amoeba (Sillo et al., 2011; Varas et al., 2018) and requires a fully functional T3SS_SPI-2_ for this process (Sillo et al., 2011). Thus, to evaluate the role played by the Tat system in the virulence of *S*. Typhimurium in *D. discoideum*, we compared the effect of feeding the amoeba with the wild-type strain or its Δ*tatABC* derivative during the development cycle, which mainly involves three sequential stages: aggregation, elevation and culmination (Figure 4A). *E. coli* B/r, routinely used to feed the amoeba during growth in the laboratory (Fey et al., 2007), and the attenuated mutant Δ*aroA* were included as controls in our assay.

As reported (Varas et al., 2018), the wild-type strain caused a delay in the social development of the amoeba where only the aggregation phase was reached after 2 days of co-incubation (Figure 4B). In contrast, the Δ*tatABC* mutant allowed the development of the amoeba until reaching the elevation phase (and in some cases the culmination phase) after 2 days of co-incubation. The same phenotype was observed in the case of the Δ*aroA* mutant and *E*. *coli* B/r. Noteworthy, the Δ*tatABC* mutant harboring plasmid pTAT caused a delayed social development of the amoeba similar to that caused by the wild-type strain, and the presence of the empty vector did not affect the phenotype shown by the Δ*tatABC* mutant (Figure 4B). It is important to mention that the ability of each bacterial strain to delay the social development of the amoeba correlated with its ability to proliferate intracellularly in this organism after 24 h of infection (compare Figures 3A, 4B).

Given that monitoring the progress of *D. discoideum* social development is used to evaluate the virulence of pathogenic bacteria (Sillo et al., 2011; Bravo-Toncio et al., 2016; Ouertatani-Sakouhi et al., 2017; Marcoleta et al., 2018; Varas et al., 2018), our results indicate that abrogation of Tat system attenuates *S*. Typhimurium in this model organism. Therefore, the Tat system contributes to *S*. Typhimurium virulence in *D. discoideum*. This is consistent with the attenuation showed by a Δ*tatC* mutant of *S*. Typhimurium in mice and murine macrophages (Santiviago et al., 2009; Reynolds et al., 2011; Craig et al., 2013), and by *tatB* and *tatC* mutants of *S*. Enteritidis in mice, chickens and Caco-2 epithelial cells (Mickael et al., 2010; Silva et al., 2012).

According to our results, the attenuation showed by the Δ*tatABC* mutant in *D. discoideum* (as revealed by its inability to delay the social development of the amoeba) cannot be explained by a diminished toxic effect exerted by this strain (for instance, caused by defective secretion of a particular exotoxin via the Tat system), as no differences in cytotoxicity were detected when *D. discoideum* was co-incubated with the wild-type strain or the Δ*tatABC* mutant under the same experimental conditions (MOI and time of infection) (Figure 2 and Supplementary Figure S2). On the other hand, the attenuation phenotype can be explained by the inability of the Δ*tatABC* mutant to survive and proliferate intracellularly in this amoeba, as revealed by our infection assay (Figure 3A). In a recent study, the role played by Tat substrates in *S*. Typhimurium virulence was studied (Craig et al., 2013). To this end, the authors inactivated every gene encoding a Tat-exported protein and evaluated the virulence of each mutant strain in a mouse model of infection. Noteworthy, no single Tat-exported substrate accounted for the strong attenuation showed by a Tat-defective mutant (Δ*tatC*) in this model. However, this attenuation was attributed to failure translocating three Tat substrates: AmiA, AmiC, and SufI. AmiA and AmiC are N-acetylmuramoyl-L-alanine amidases, and SufI (also named FtsP) is involved in bacterial division. Therefore, the attenuation of a Tat-defective mutant of *S*. Typhimurium in the mouse model of infection was associated with failure to translocate Tat substrates linked to the cell division machinery, resulting in envelope defects (Craig et al., 2013). Most probably, the attenuation and intracellular survival and proliferation defects shown by the Δ*tatABC* mutant in *D. discoideum* can be explained by these envelope defects. Further studies are required to confirm this hypothesis.

## Conclusion

Overall, our results indicate that the Tat system is essential for *S*. Typhimurium to survive and proliferate intracellularly in *D. discoideum* and for virulence in this host. To the best of our knowledge, this is the first report on the relevance of the Tat system in the interaction of any bacterial pathogen with the social amoeba *D. discoideum*.

## Author Contributions

ÍU, AS, CV, and CS conceived and designed the experiments, and analyzed the data. ÍU, AS, BL, and CV performed the experiments. ÍU, CV, SÁ, and CS contributed reagents, materials, and analysis tools, and wrote the paper. All authors read and approved the final manuscript.

## Conflict of Interest Statement

The authors declare that the research was conducted in the absence of any commercial or financial relationships that could be construed as a potential conflict of interest.
