# Supplementary material for: Contribution of the Twin-Arginine Translocation System to the Intracellular Survival of Salmonella Typhimurium in Dictyostelium discoideum
Source: Front Microbiol. 2018 Dec 6;9:3001. doi: 10.3389/fmicb.2018.03001 (PMC6291500; doi:10.3389/fmicb.2018.03001)
Supplement: Supplementary file 1 [file Data_Sheet_1.PDF]

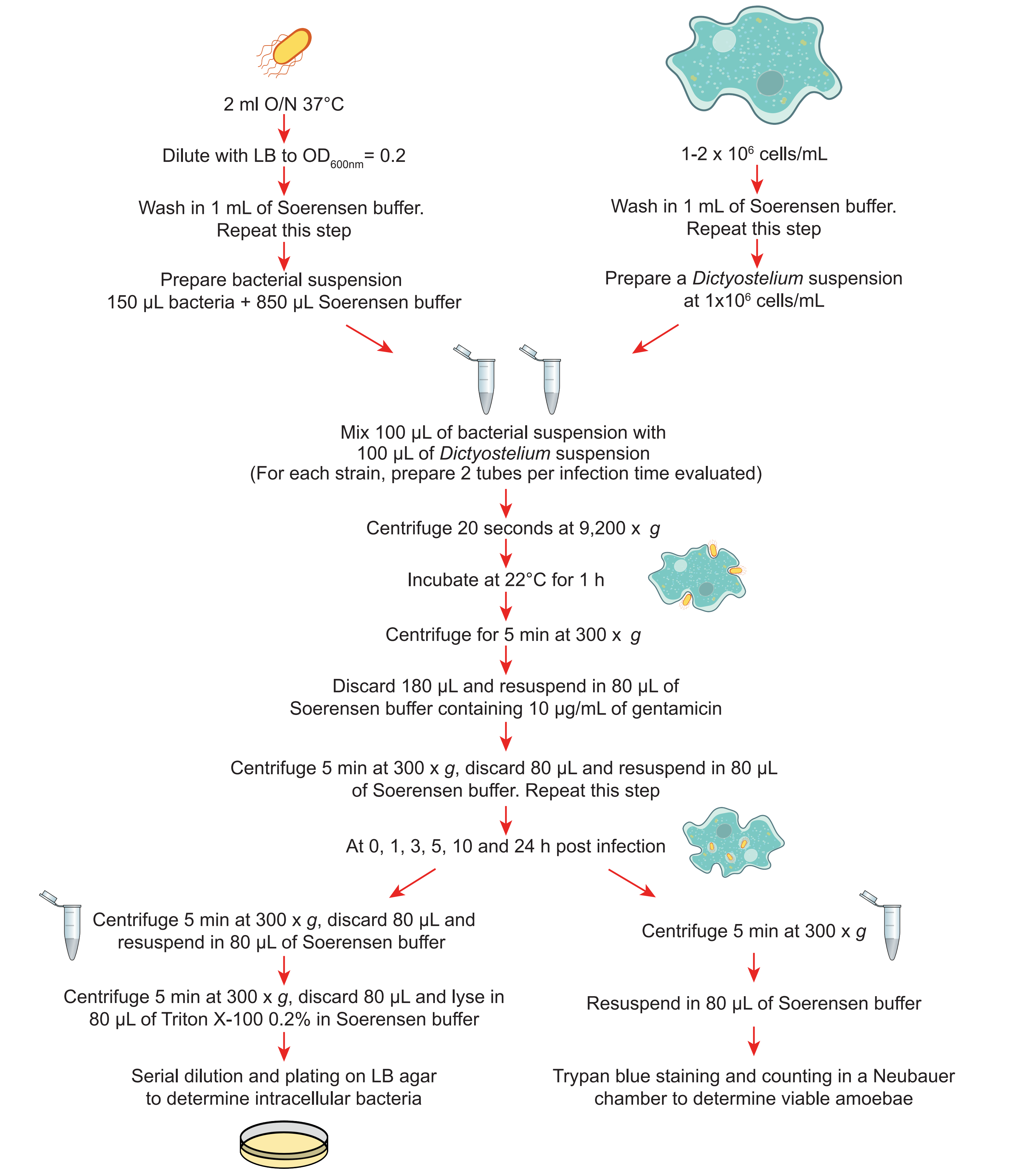


**Supplementary Figure S1.- Infection assay to evaluate internalization and intracellular survival in *D. discoideum*.** The Materials and Methods section in the manuscript includes a detailed description of the procedure.
