# Supplementary material for: Contribution of the Twin-Arginine Translocation System to the Intracellular Survival of Salmonella Typhimurium in Dictyostelium discoideum
Source: Front Microbiol. 2018 Dec 6;9:3001. doi: 10.3389/fmicb.2018.03001 (PMC6291500; doi:10.3389/fmicb.2018.03001)
Supplement: Supplementary file 2 [file Data_Sheet_2.PDF]

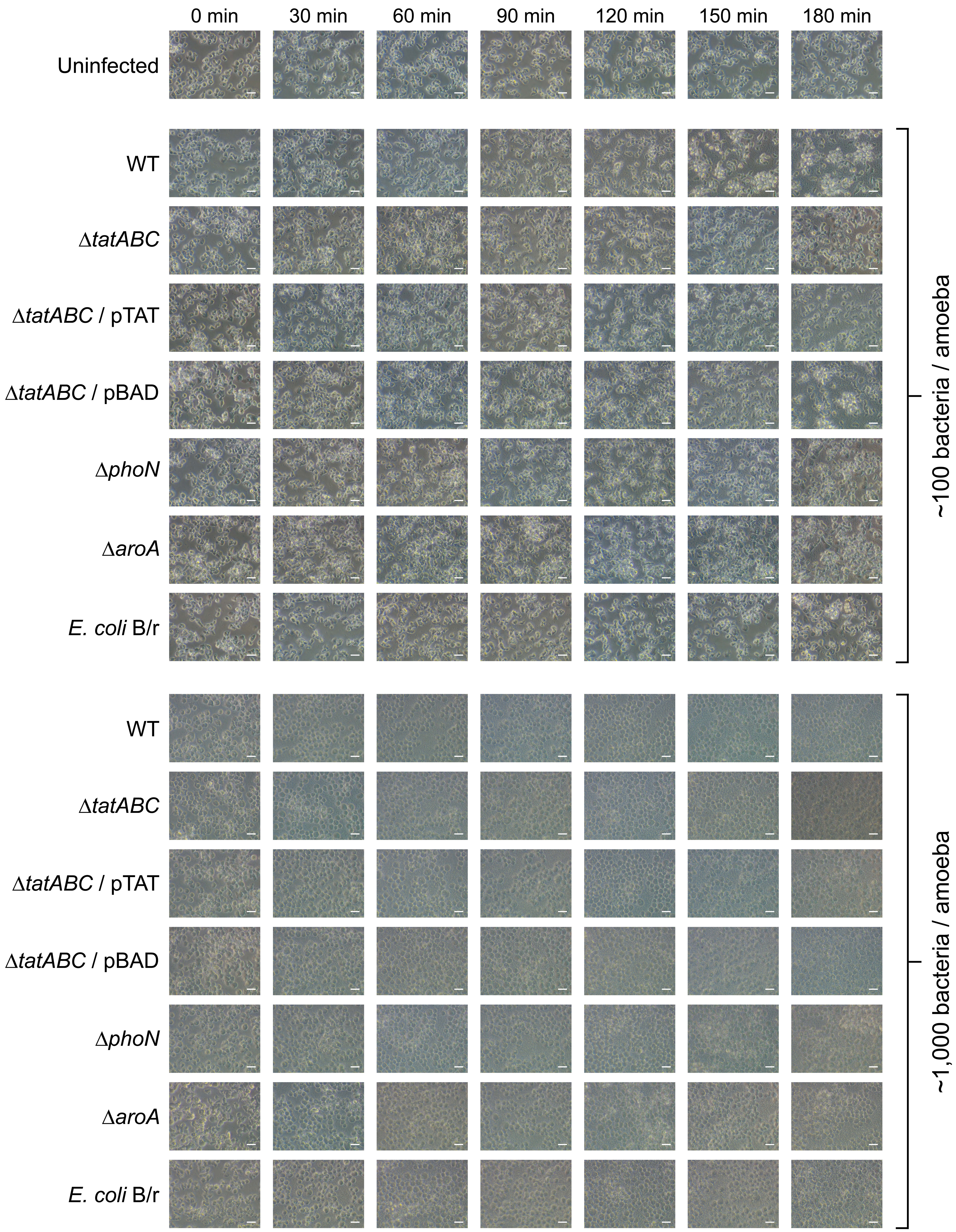

Supplementary Figure S2.- Qualitative evaluation of bacterial cytotoxicity on *D. discoideum*. The shape of *D. discoideum* AX4 cells infected with different strains of *S*. Typhimurium or *E. coli* B/r was monitored at 0, 30, 60, 90, 120, 150 and 180 min of co-incubation at 22°C in Soerensen buffer. Representative images from 3 independent assays carried out at MOIs of ~100 or ~1,000 bacteria/amoeba are shown. Scale bar, 20 µm.
